# Haplotype networks of SARS-CoV-2 infections in the *Diamond Princess* cruise ship outbreak

**DOI:** 10.1073/pnas.2006824117

**Published:** 2020-07-28

**Authors:** Tsuyoshi Sekizuka, Kentaro Itokawa, Tsutomu Kageyama, Shinji Saito, Ikuyo Takayama, Hideki Asanuma, Naganori Nao, Rina Tanaka, Masanori Hashino, Takuri Takahashi, Hajime Kamiya, Takuya Yamagishi, Kensaku Kakimoto, Motoi Suzuki, Hideki Hasegawa, Takaji Wakita, Makoto Kuroda

**Affiliations:** ^a^Pathogen Genomics Center, National Institute of Infectious Diseases, 162-8640 Tokyo, Japan;; ^b^Influenza Virus Research Center, National Institute of Infectious Diseases, 162-8640 Tokyo, Japan;; ^c^Department of Virology 3, National Institute of Infectious Diseases, 162-8640 Tokyo, Japan;; ^d^Infectious Disease Surveillance Center, National Institute of Infectious Diseases, 162-8640 Tokyo, Japan;; ^e^National Institute of Infectious Diseases, 162-8640 Tokyo, Japan

**Keywords:** SARS-CoV-2, COVID-19, *Diamond Princess*, haplotypes

## Abstract

The *Diamond Princess* cruise ship was put under quarantine offshore Yokohama, Japan, after a passenger who disembarked in Hong Kong was confirmed as a coronavirus disease 2019 case. We performed whole-genome sequencing of severe acute respiratory syndrome coronavirus 2 (SARS-CoV-2) directly from PCR^+^ clinical specimens and conducted a phylogenetic analysis of the outbreak. All tested isolates exhibited a transversion at G_11083_T, suggesting that SARS-CoV-2 dissemination on the *Diamond Princess* originated from a single introduction event before the quarantine started. Although further spreading might have been prevented by quarantine, some progeny clusters could be linked to transmission through mass-gathering events in the recreational areas and direct transmission among passengers who shared cabins during the quarantine. This study demonstrates the usefulness of haplotype network/phylogeny analysis in identifying potential infection routes.

## Significance

On February 5, 2020, the *Diamond Princess* cruise ship was put under quarantine offshore Yokohama, Japan, after a passenger who disembarked in Hong Kong was confirmed to have coronavirus disease 2019. Whole-genome sequencing of severe acute respiratory syndrome coronavirus 2 (SARS-CoV-2) and a network/phylogeny analysis of the outbreak suggested that there was a single introduction of SARS-CoV-2, which disseminated among passengers on the ship through possible mass-gathering events in the recreational areas. These findings provide information useful for preventing future outbreaks on cruise ships and emphasize the importance of genome-based network/phylogeny analysis to elucidate possible transmission routes in outbreak situations.

In late December 2019, an outbreak of a novel coronavirus disease 2019 (COVID-19) originated in Wuhan, China. It was caused by a new strain of betacoronavirus: Severe acute respiratory syndrome coronavirus 2 (SARS-CoV-2) (1, 2). The *Diamond Princess* (DP) cruise ship was put under quarantine soon after its return to Yokohama, Japan on February 3, 2020, after visiting Kagoshima, Hong Kong, Vietnam, Taiwan, and Okinawa, because an 80-y-old passenger who disembarked in Hong Kong was confirmed as a COVID-19 case on February 1 after the ship had departed from Hong Kong on January 25. The passenger presented with a cough beginning on January 23. From February 3 to 4 the health status of all passengers and crew members was investigated by quarantine officers and upper-respiratory specimens were collected from symptomatic passengers, crew, and their close contacts for SARS-CoV-2 PCR testing. As of February 5, there were a total of 3,711 individuals with 2,666 passengers and 1,045 crew members on board the DP. The Japanese government asked about 3,600 passengers and crew members to stay on board in Yokohama during the 14-d isolation period through February 19 to prevent the further spread of COVID-19 cases. A field epidemiological study about the DP COVID-19 cases has been published (3). As of March 8, 697 COVID-19 cases had been identified among the 3,711 persons on the DP and 7 people had died (4).

Here, we have generated a haplotype network of the SARS-CoV-2 outbreak using genome-wide single nucleotide variations (SNVs), identifying the genotypes of isolates that disseminated in the DP cruise ship after quarantine on February 5, 2020.

## Results and Discussion

During the period from February 15 to 17, 2020, we obtained pharyngeal specimens of 896 persons (880 passengers, 15 crew members, and 1 quarantine officer). A quantitative reverse-transcription PCR (RT-qPCR) test for SARS-CoV-2 was positive for 148 individuals (138 passengers, 9 crew members, and 1 quarantine officer), indicating that the positivity rate was 16.4% (147 positive individuals [excluding the officer] of 895 subjects). This positivity rate was comparable to the rate determined after finishing PCR testing for all persons [619 PCR positives for 3,711 persons, 16.6%; see National Institute of Infectious Diseases Field Briefing report for more detail (5)].

**Fig. 1.**
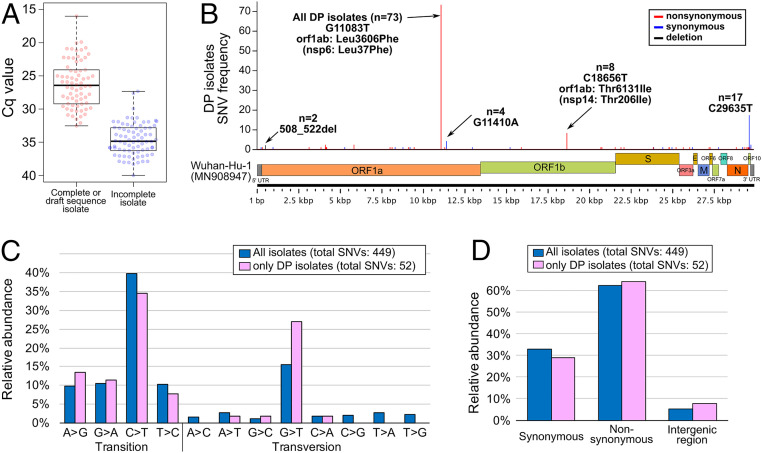
WGS of SARS-CoV-2 isolates (strains) on the DP cruise ship off Yokohama in Japan. (*A*) Box plots of the Cq values from RT-qPCR assays that either successfully resulted in WGS or incomplete sequencing. A Cq of at least 32 (∼300 viral copy) is favorable for obtaining a whole-genome sequence by PrimalSeq. (*B*) The genome position of genetic variations found among DP isolates compared with the isolate Wuhan-Hu-1 (MN908947). (*C*) The detected nucleotide transition and transversion in all GISAID available SARS-CoV-2 genomes (*n* = 412, isolation date by February 19, 2020; updated on May 14, 2020) (Dataset S3) and DP isolates (*n* = 73) compared with the isolate Wuhan-Hu-1 (MN908947). (*D*) The detected ratio of synonymous/nonsynonymous mutations on coding sequences or intergenic regions in all GISAID available SARS-CoV-2 genomes is described above (*n* = 412) and DP isolates (*n* = 73) compared with the isolate Wuhan-Hu-1 (MN908947).

A detected cycle quantification (Cq) value of RT-qPCR for the 896 specimens is shown in Dataset S1. All 148 RT-qPCR positive RNA samples were subjected to the PrimalSeq protocol to generate enriched cDNA of the SARS-CoV-2 genome, followed by next-generation sequencing (NGS). In total, 70 whole-genome sequences have been determined. The Cq limit for successful genome sequence determination was around 32 (Fig. 1*A*), which corresponds to a virus copy number of less than 300. The 73 genome sequences (including 3 sequences of DP isolates deposited in the Global Initiative on Sharing All Influenza Data [GISAID]) were compared with the Wuhan-Hu-1 (isolated on December 26, 2019 in China) genome sequence as a reference. The frequencies of SNVs suggested that all 73 isolates shared a SNV: The G nucleotide at the 11,083 position on the Wuhan-Hu-1 genome sequence was mutated to T (G_11083_T transversion), leading to a nonsynonymous amino acid substitution (Leu_37_Phe) in the nsp6 protein (Fig. 1*B*). Some additional minor SNVs were identified throughout the genome sequence.

A number of nucleotide variations have been identified; specifically, 52 SNVs among DP isolates (*n* = 73) and 449 SNVs among all tested isolates, including entries from GISAID (*n* = 412), (Dataset S3) (Fig. 1*C*). Pyrimidine transition, in particular cytosine (C) to thymine (T), makes up a large portion of the SNVs, suggesting that hydrolytic deamination of cytosine and 5-methylcytosine residues in DNA appears to contribute significantly to the appearance of spontaneous mutations (6). In contrast, a relatively low frequency of transversion mutations was observed, but the guanine (G) to thymine (T) transversion in particular has been observed in both DP isolates and other SARS-CoV-2 isolates (Fig. 1*C*). The occurrence of nonsynonymous mutations (62%) was apparently higher than that of synonymous mutations (33%) and mutations in noncoding/intergenic regions (5%) (Fig. 1*D*).

**Fig. 2.**
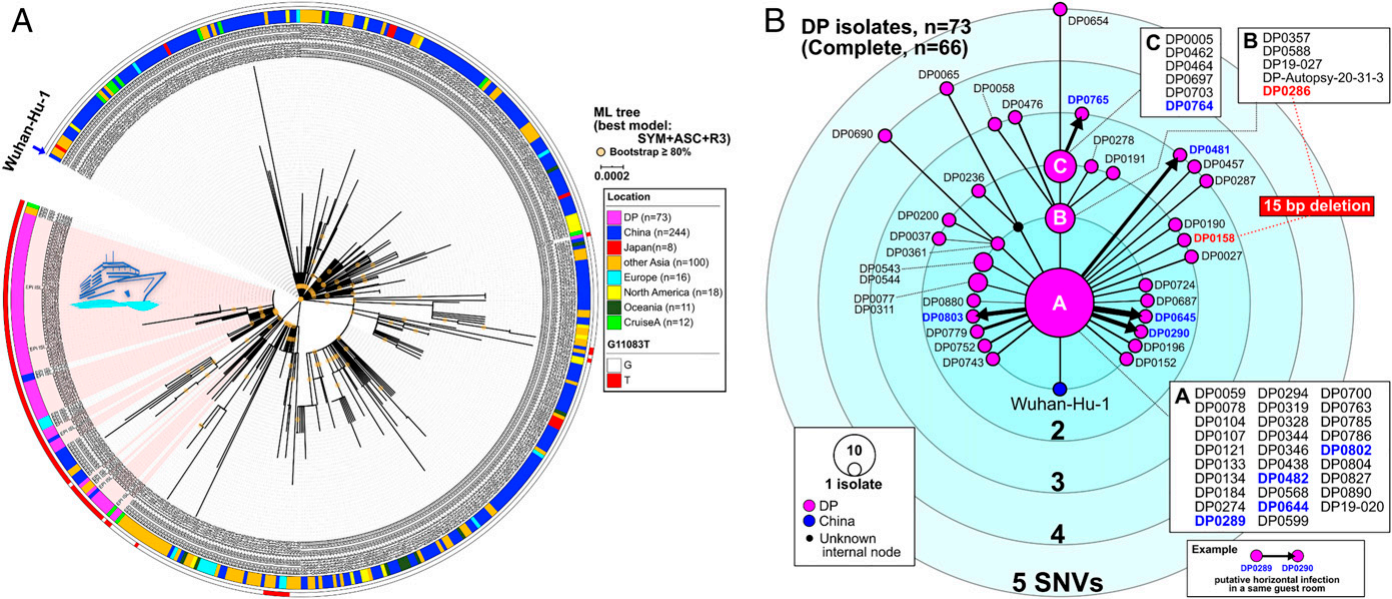
Haplotype network using genome-wide SNVs (HN-GSNVs). (*A*) An ML phylogenetic analysis was conducted using 73 SARS-CoV-2 isolates (February 15 to 17, 2020) from the DP cruise ship off Yokohama in Japan compared with all GISAID-available SARS-CoV-2 genomes (*n* = 412, isolation date by February 19, 2020; updated on May 14, 2020). In total, 447 SNVs were detected in 482 isolates. The isolate location and G11083T allele information are described in the color schemes on the outside of the phylogenetic tree. (*B*) Clusters of the DP isolates (*n* = 73) and Wuhan-Hu-1 were compared with a median-joining SNV network analysis to identify the strain that was first spread (indicated A, *n* = 29 isolates) and subsequent transmissions (indicated B, *n* = 5 isolates; indicated C, *n* = 6 isolates; and the other 33 isolates). Information on each isolate can be obtained in Dataset S1. A possible transmission between partners in the same cabin after the quarantine is shown by thick arrows between isolate nodes.

Maximum-likelihood (ML) phylogenetic analysis including other SARS-CoV-2 genome sequences that are publicly available on GISAID and haplotype networks from genomic SNVs (HN-GSNVs) were used to map the genotypes of the SARS-CoV-2 isolates that disseminated in the DP cruise ship after isolation of the passengers on February 5, 2020 (Fig. 2). The ML tree indicated that three European isolates are closely located in DP clusters; however, these three isolates from Paris (EPI_ISL_408430, EPI_ISL_410984, and EPI_ISL_411218) were collected on January 29 or February 2, 2020, which was before the time of collection of the DP isolates after Yokohama offshore quarantine on February 5, 2020. This suggests that Wuhan-related isolates had already spread in Europe in an independent event earlier than the DP cruise ship outbreak. Thus, we speculated that the ML tree might mislead the tracing of a possible infectious route based on genetic alterations. To decode the genealogies of the whole genome of SARS-CoV-2, we generated HN-GSNVs to highlight the clonality of DP isolates. Analysis clearly suggested that the DP cluster had been generated from the potential haplotype ancestor of the global pandemic, Wuhan-Hu-1 haplotype, by G_11083_T transversion before initial dissemination in the DP (Fig. 2*B*; see Dataset S2 for SNVs). The finding that all isolates in the DP cluster exhibit G_11083_T transversion strongly suggest that SARS-CoV-2 dissemination in the DP could have originated from a single introduction event before the quarantine started on February 3.

Further focusing of the HN-GSNVs map on only the DP isolates in the cluster revealed that the 29 isolates of the DP-A cluster (indicated A in Fig. 2*B*) predominated among the DP isolates, indicating that the DP-A cluster is the ancestral haplotype for subsequent transmission. The estimated evolutionary rate of SARS-CoV-2, described on the Nextstrain website (https://nextstrain.org/ncov?l=unrooted, last updated on May 15, 2020), of 24.17 substitutions per genome per year on average suggests that predominant isolates in the DP-A cluster could be a very early generation of progeny from Wuhan-Hu-1 isolated on December 26, 2019. This predomination of the DP-A cluster might explain the large epidemic within the cruise ship; it is possible that superspreading occurred immediately after introduction of the virus, well before the quarantine started on February 3, 2020. Although further explosive spreading might have been prevented after the quarantine, some of the subsequent progeny clusters, including DP-B (five isolates) and DP-C (six isolates) (Fig. 2*B*), may have formed via transmission through hidden links, such as eating at the same dinner table. Additionally, 33 patients (45%) not included in the DP-A, -B, and -C clusters were revealed to have unique SARS-CoV-2 haplotypes with patient-specific unique SNVs and/or deletions. The finding of such unique progenies might explain how further subsequent minor spreading occurred after the quarantine. Indeed, possible direct transmissions among passengers who shared the same cabins during the quarantine have been speculated; three cabin mates showed infection by viruses with the same haplotype (two pairs in the DP-A cluster and one pair in another cluster), and five pairs shared haplotypes linked by a single SNV (A to DP0290, A to DP0481, A to DP0645, A to DP0803, and DP0764 to DP0765).

We did not find any area-specific events of COVID-19 spread in the DP because all COVID-19 patients were widely distributed in all 18 decks across the ship. This may indicate that most SARS-CoV-2 infections began at mass-gathering events in the recreational areas, where all passengers enjoyed dancing, singing, shopping, and watching performances. The highest incidence of COVID-19 onset was observed on February 7, 2020; the epidemic curve in the latter half of the epidemic was dominated by crew members whose work was not strictly controlled to maintain service on the ship (7).

Nishiura (7) suggested that the peak time of infection was from February 2 to 4, predicted by back-calculating the incidence of SARS-CoV-2 infections on the DP, and that the incidence abruptly declined afterward. Nishiura also stated that the estimated incidence indicates that the movement restriction policy was highly successful in greatly reducing the number of secondary transmissions on board the DP. Another report by Rocklöv et al. (8) suggested that the public health measures and quarantine prevented more than 2,000 additional cases compared to a scenario in which no interventions were taken. Rocklöv et al. did acknowledge that evacuating all passengers and crew early would have prevented many more passengers and crew from becoming infected, although this containment method was not realistic at the time. Zhang et al. (9) also estimated that the reproduction number (*R*_0_) for COVID-19 cases was 2.28 (95% confidence interval: 2.06 to 2.52) during the early stage, and estimated that the outbreak size on the DP cruise ship might have been greater, if the strict infection management and quarantine had not been conducted.

An increased number of COVID-19 cases has been confirmed, and it is now difficult to identify infection routes because some Japanese cases have no recent travel history to China or contact with persons from Wuhan City or Hubei Province in China. Regarding the quarantine on the DP cruise ship, the enclosed circumstances revealed how SARS-CoV-2 can be spread in the community. Further field epidemiological studies should be conducted to trace the link of infections but, unfortunately, efforts to identify the route of transmission are still limited. This study demonstrated that HN-GSNVs can contribute to epidemiologic field investigations and identified possible transmission routes in the DP cruise ship outbreak after the quarantine was enacted.

## Materials and Methods

### Clinical Specimens and RT-qPCR Testing for COVID-19 Infection.

An RT-qPCR test for SARS-CoV-2 (10, 11) was performed to allow passengers to disembark. A Cq value in RT-qPCR was determined using 10-fold serial dilutions of a synthesized control RNA (the N gene) from 5 to 5,000 copies (10). We obtained the pharyngeal specimens of 896 persons (880 passengers, 15 crew members, and 1 quarantine officer) from the DP, taken on February 15 to 17, 2020 before disembarking on February 19, 2020. This study was approved by the research ethics committee of the National Institute of Infectious Diseases (approval no. 1091). It was conducted according to the principles of the Declaration of Helsinki, in compliance with the Law Concerning the Prevention of Infections and Medical Care for Patients of Infections of Japan. The ethical committee waived the need for written consent regarding the research into the viral genome sequence. The personal data related to the clinical information were anonymized, and our procedure is not to request written consent for all patients suffering from COVID-19.

### Whole-Genome Sequencing of SARS-CoV-2.

To elucidate the route of dissemination on the DP during the quarantine period, we conducted whole-genome sequencing (WGS) of SARS-CoV-2 using a multiplex PCR-based RNA sequencing (PrimalSeq) (12, 13) technique directly from PCR^+^ clinical specimens. WGS analysis provides useful information for tracing back infection linkages. However, the virus content in pharyngeal swabs is not abundant at the early onset of COVID-19, which might lead to difficulties in achieving WGS. Indeed, the genome size of SARS-CoV-2 is 29.9 kb, which is the largest among known RNA viruses, suggesting that some improvements will be required to enrich the pathogen RNA molecule at every preparation step of the sequencing protocol to efficiently carry out WGS.

Whole-genome sequences of SARS-CoV-2 isolates were obtained by the PrimalSeq protocol for producing enriched cDNA of the SARS-CoV-2 genome by multiplex RT-PCR amplicons using a multiplexed PCR primer set, which was proposed by the Wellcome Trust ARTIC Network (14). The PCR products from the same clinical samples were pooled, purified, and subjected to Illumina library construction using the QIAseq FX DNA Library Kit (Qiagen). The NextSeq 500 platform (Illumina) was used for sequencing the indexed libraries. We found that two particular amplicons regularly showed low to zero coverage due to primer dimerization as described in Itokawa et al. (15) (we should note that the modified primer was not used in this study). Then, we used data from another round of 3-kb RT-PCR assays covering the whole genome of SARS-CoV-2 (29.9 kb) with 10 PCR amplicons. The 3-kb RT-PCR multiplexed amplicons were mixed in a single tube and subjected to shotgun NGS on the NextSeq 500 platform (Illumina) as described for the ARTIC library. Thirty nucleotides corresponding to the multiplex primer sequences at both ends of NGS reads were trimmed for further analysis. The obtained NGS reads were mapped to the SARS-CoV-2 Wuhan-Hu-1 reference genome sequence (29.9 kb single-strand RNA; GenBank ID: MN908947), resulting in a specimen-specific SARS-CoV-2 genome sequence fully mapped with a read depth of at least ≥100× from the 99- to 29,796-nt region of Wuhan-Hu-1 genome sequence (see details in Dataset S2). The full genome sequence was determined by de novo assembly using A5-miseq v.20140604 (16).

### Comparative Genome-Sequence Analysis and SNV Analysis.

The nearly full-length genome sequence (≥29 kb) of SARS-CoV-2 isolates until February 19, 2020 were retrieved from the GISAID EpiCoV database on May 14, 2020, followed by multiple alignment using MAFFT v7.222 (17). The poorly aligned regions in 5′ and 3′ ends were trimmed; we determined that the core regions were from the 99- to 29,794-nt position by comparison against the Wuhan-Hu-1 genome sequences (GISAID ID, EPI_ISL_402125; GenBank ID, MN908947.3). Gap-containing sequences in the core region were excluded; sequences of 243 isolates from the GISAID database were used in subsequent analyses. An ML phylogenetic analysis with SNVs was performed using iqtree v1.6.12 (18) with an ultrafast bootstrap test parameter, followed by visualization using iTOL v4 (19). The genome sequences of the DP isolates were aligned using MAFFT with sequences retrieved from the database, followed by extraction of SNV and deletion sites. The SNV median-joining network analysis was performed using the PopART software (20).

## Supplementary Material

Supplementary File

Supplementary File

Supplementary File

## Data Availability

Whole-genome sequence (fasta) data have been deposited in the Global Initiative on Sharing All Influenza Data database (GISAID; https://www.gisaid.org/epiflu-applications/next-hcov-19-app/) with accession IDs EPI_ISL_416565 to EPI_ISL_416634 (Dataset S1) and GenBank under accession nos. LC570961–LC571041.
